# On Intra-Uterine Goitre, or Bronchocele

**Published:** 1856-01

**Authors:** 


### *On Iutra-Uterine Goitre, or Bronchocele.*

Various kinds of tumor in the cervical region of the fœtus have been found at the time of birth.

1. The cervical portion of the vertebral column is sometimes, though not so often as the loins or back, the seat of spina bifida; and the resulting tumor has been seen to vary in size from the bulk of a nut to that of the infant's head.

2. Meckel, Otto and several other pathologists have described a variety of congenital cystic tumor of the cellular tissue, situated at the posterior part of the neck, and remarkable for a central pillared division, into two lateral and symmetrical lobes, by the ligamentum nuchæ. I have seen it mistaken for a spina bifida.

3. Numerous instances of intra-uterine tumors in the anterior and lateral portions of the cervical region have been recorded of late years by

Berndt, Cæsar Hawkins, Beatty, Mutter and others, under the name of cysts, or "congenital hydrocele" of the neck. These tumors, which sometimes grow much after birth, usually consist of one, two or more large serous cysts, capable of being emptied and obliterated by puncture and the injection of iodine ; by setons, &c. Sometimes an agglomeration of small cysts enters also into their composition.

4. A kind of large cystic tumor, the mass of which consists of a numerous collection of small cells filled with thickish glairy fluid, is occasionally observed at birth in the upper part of the neck, and projects more or less into the mouth. It seems to be a true ranula, originating and consisting of hypertrophy and enlargement of the salivary glands. I have seen two instances of it where the children died a few days after birth, the puncturing of one or two cells being of no use in diminishing the mass. There is a specimen of it in the obstretic museum of the University, forming a large tumor at birth. This form of apparent cystiform tumor, has I believe, been frequently confounded with the third variety alluded to above.

5. Among the tumors on the neck at birth, I have seen one remarkable instance of a large flattish swelling on the posterior cervical region, covered with skin of the usual color and appearance, and formed of a deep-seated erectile vascular tissue, which in a great measure disappeared under pressure, and enlarged when the child cried or strained. I treated it by various means, none of which produced complete obliteration. Some years afterwards I heard it was cut out by a surgeon, and the resulting hemorrhage was most excessive.

6. Few cases of congenital enlargement of the thyroid gland, or of true intra-uterine goitre, or bronchocele, have hitherto been placed upon record. The following cases, however, will show that goitre constitutes one form of cervical tumor, which may be occasionally met with at birth.—Bronchocele is sometimes hereditary, but very few instances of it have been at birth in infants so predisposed. Usually there is no trace of it till some years subsequently. The following is the only exception to this general remark which I have been able to find:

Case 1.—In an essay on goitre, published in 1824, M. Ferus refers to a congenital instance of the disease, which had occurred in the practice of M. Godelle, physician to the Hospital of Soissons, and where the mother was affected with the same disease.* The child only survived for a few hours after birth.†

*Dictionnaire de Medecine, vol. x., p. 283.

†In the Archives Generales de Medecine, vol. xiii., p. 76, Dr. Casau speaks of a remarkable case of hereditary goitre, where a young infant in the family died of it; but whether it was hereditary or not in this child, is not precisely stated. "A woman, aged 23 years, married, affected with pulmonary consumption in the second degree, presented to us an example of the obstinate hereditary predisposition of pulmonary phthisis and of goitre; her young infant, (*jeune infant*) her father, and seven brothers of her father had died of the former disease; one of her paternal aunts, who showed no disposition to phthisis, carried a very large goitre; herself (the patient) was affected with goitre, which had considerably diminished since the first symptoms of phthisis had been developed. All her brothers and sisters had been victims to that cruel affection; only one sister, who had goitre commencing, enjoyed good health at that period. One could say in that family, that the two affections were in such relation, that the one appeared reciprocally to supplant the other."

Lately I met with a marked instance of intra-uterine goitre in my own practice, and had an opportunity of ascertaining its true nature by dissection.

Case 2.—The mother of the child never suffered from any symptoms of goitre, or lived in any place where the disease was endemic. She has now borne ten children. The first seven of these infants were stillborn. They all died, I believe from reports given to me, of disease of the placenta, and not from any malady of malformation in their own bodies.—During her last four pregnancies she has been under my professional care, and has always taken, in the latter periods of utero-gestation, large and continuous doses of chorate of potass. The four last children were born alive, and have continued to live, with the exception of the last, namely, the one born with goitre. It survived only for about eight hours after birth, and would have died much earlier from asphyxia if a catheter had not been retained in the trachea to obviate the compression of the mass of bronchocele. The child was born two or three weeks before the full term, labor having been induced in consequence of the child's heart beginning to beat with morbid slowness. The goitrous enlargement of the thyroid gland was nearly of the size of a hen's egg. It rendered the labor tedious, by preventing—as the hands or arms placed in the hollows of the neck sometimes do—the proper flexion of the head, and the approach of the chin to the sternum; the presentation in consequence being one of the forehead and not of the parietal bone. The goitre, or bronchocele, as seen after birth, appeared to fill up entirely the space or hollow between the chin and sternum. On examination after death, it was found to surround almost entirely, and compress, the trachea. All parts of the thyroid gland were equally affected. The goitrous tumor was comparatively smooth on its surface, but had a small, irregular nodule attached anteriorly to its upper border, close to the body of the hyoid bone.

Internally, it presented a firm, glandular structure, and under the microscope, it appeared to consist of the usual thyroid tissues, greatly hypertrophied. The vesicular cavities of the gland seemed not only increased in number, but enlarged in size also, and the septa within them were thickened. They were distended with epithelial contents. The external surface of the brain of the child was surrounded with a large quantity of serum, and the brain itself was considerably below the usual size. The opening of the eyelids was almost small. The thymus gland, supra-renal capsules, etc., were normal in size and structure; and there was no other unusual appearance detected. In his essay on the pathological anatomomy of new-born infants,* Dr. F. Weber describes an example of congenital goitre, similar in several respects to the preceding instance.

Case 3.—A child was born some weeks before the ninth month, and it survived only a few minutes. The goitrous thyroid gland projected forward in the cervical region, was about half an inch thick, and extended not only laterally, but also backward, and some distance over the upper part of the trachea, though not to such a degree that a union of both lateral lobes had occurred posteriorly. On being cut into, the parenchyma of the bronchocle appeared dark red, and the microscope showed within it a quantity of effused blood-globules, which were not evident to the naked eye. In other respects, the parenchyma of the tumor presented internal-

---

*Beitrage zur Path. Adatemie der Neugebornen, p. 84.

ly the normal structure of the thyroid gland. The thymus gland appeared also larger than usual, and particularly on one side, but without any change of sructure. There was a considerable degree of hydrocephalus present, with contraction of the extremities.

CASE 4.—When describing the case No. 2 to the Medico-Chirurgical Society, immediately after the time of its occurrence in February, 1855,* Dr. Keiller stated that he had, a few months previously, met with an instance of the same disease, where the child's head at birth offered the same unusual presentation. I have lately examined the child, who is now about a year old, with Dr. Keiller. There is still a large, irregularly-lobulated swelling in the region of the thyroid gland, and stretching somewhat upward on each side of the trachea. It projects forward, and appears to swell out when the child cries. At other times, the skin of the neck looks flaccid, wrinkled and empty, over the site of the tumor, in consequence of the tumor itself having diminished and shrunk considerably since the time of birth. The lobulated masses of the tumor feel firm and hard to the touch; and probably the intervening and connecting tissue, in which the absorption has been specially marked, was originally more cystic in its character. The tumor does not seem to affect in any way the general health and growth of the child. The mother was born and brought up in the county of Cumberland, where goitre is not uncommon; but neither she nor any of her relatives were ever in the least degree affected by it. The present goitrous infant is the first child which she has borne. Before pregnancy occurred, she was under my care for chronic metritis; but her general health was good.

An instance of congenital cervical tumor, under the title of "Scrofula in Fœtu," was long ago described by Francus,† with characters and a site which have made Grætzer and Montgomery refer it to the head of goitre. In this, as in Dr. Keiller's case, the certainty of the tumor consisting of enlargement of the thyroid gland was not made out by dissection.

CASE 5.—The child—a boy—presented at birth a tumor on both sides of the neck, but it was largest on the left. When the infant, cried, or moved his neck too freely, that on the left side swelled excessively, and appeared to interfere with the power of suction and delutition. Francus adds, that he unsuccessfully tried to effect the removal of the swelling by various remedies, local and general, and that notwithstanding it increased daily in size.—*Ib.*

